# Supplementary material for: Narrowband, angle-stable, and highly efficient polariton organic light emitting diodes employing thermally activated delayed fluorescence
Source: Light Sci Appl. 2026 Sep 14;15:378. doi: 10.1038/s41377-026-02415-1 (PMC13572423; doi:10.1038/s41377-026-02415-1)
Supplement: Supplementary file 1 [file 41377_2026_2415_MOESM1_ESM.docx]

**Supplementary Information for: Narrowband, angle-stable, and highly efficient polariton organic light emitting diodes employing thermally activated delayed fluorescence**

Andreas Mischok^1,#,^*, Simon Lennartz^1,#^, Vanessa Gruber^1^, Francisco Tenopala-Carmona^1^, Julia Witt^1^, Sabina Hillebrandt^1^, Malte C. Gather^1,2,^*

*^1^Humboldt Centre for Nano- and Biophotonics, Department of Chemistry and Biochemistry, University of Cologne, Greinstr. 4-6, 50939 Köln, Germany*

*^2^School of Physics and Astronomy, University of St Andrews, North Haugh, St Andrews KY16 9SS, United Kingdom*

*^#^These authors contributed equally.*

**contact: andreas.mischok@uni-koeln.de, malte.gather@uni-koeln.de*

**Table of Contents**

| **Figure S1** | **Current density-voltage-luminescence (j-V-L) characteristics of reference OLED, OLED with doped HTL and P-OLED Variation 4** |
| --- | --- |
| **Figure S2** | **Influence of silver anode thickness on emission linewidth** |
| **Figure S3** | **Evolution of polariton modes and angle-resolved electroluminescence spectrum when increasing cavity thickness in bottom-emitting design** |
| **Figure S4** | **Evolution of polariton modes and angle-resolved electroluminescence spectrum when increasing cavity thickness in top-emitting design** |
| **Figure S5** | **Emission spectra of polariton OLEDs at different operating voltages** |
| **Figure S6** | **Angle-resolved emission intensity of all devices** |
| **Figure S7** | **Simulated Outcoupling Efficiency of POLEDs V1** |

**Figure S1. Current density-voltage-luminescence (j-V-L) characteristics of reference OLED, OLED with doped HTL and P-OLED Variation 4.** The measured current density is not significantly influenced by the introduction of the 150-nm thick p-doped HTL into the OLED stack. Likewise, the current density-voltage characteristics for P-OLED design Variation 4, which comprises a p-doped HTL-SCL containing C545T, is also similar to the reference OLED, confirming the good electrical performance of this stack design. All three devices also reach similar luminance levels across the investigated voltage range. Reference OLED stack: ITO (anode) | 50 nm NPB (HTL) | 10 nm TCTA (EBL) | 30 nm CBP:10wt% CzDBA | 40 nm TmPyPB (ETL) | 1 nm LiF (EIL) | 100 nm Al (cathode). Doped HTL stack: ITO (anode) | 150 nm SpiroTTB:F6TCNNQ(4%) | 50 nm NPB (HTL) | 10 nm TCTA (EBL) | 30 nm CBP:10wt% CzDBA | 40 nm TmPyPB (ETL) | 1 nm LiF (EIL) | 100 nm Al (cathode). P-OLED V4 stack: 1 nm Al | 20 nm Ag (Anode) | 136 nm SpiroTTB:C545T(50%):F6TCNNQ(4%) | 50 nm NPB (HTL) | 10 nm TCTA (EBL) | 30 nm CBP:10wt% CzDBA | 40 nm TmPyPB (ETL) | 1 nm LiF (EIL) | 100 nm Al (cathode).

**Figure S2. Influence of silver anode thickness on emission linewidth.** Electroluminescence intensity spectrum under normal incidence of different bottom-emitting OLED designs. The ITO reference OLED shows a FWHM of 86 nm and a weakly coupled microcavity comprising a 20 nm silver anode shows a FWHM of 40 nm. In P-OLEDs, the linewidth is further reduced and depends on the thickness of the silver anode. P-OLEDs with an anode thickness of 20 nm, 25 nm and 35 nm shown an emission FWHM of 26 nm, 24 nm, and 20 nm, respectively.

**Figure S3. Evolution of polariton modes and angle-resolved electroluminescence spectra when increasing cavity thickness in bottom-emitting design.** Measured angle-resolved electroluminescence spectrum of P-OLEDs similar to design Variation 4, with a triple-mixed doped strong coupling layer, on false-colour scale. The HTL thickness and thus total cavity thickness increases from left to right. The lines indicate the calculated position of the cavity mode (grey dashed lines), excitons (X; grey dash–dotted lines), as well as upper and middle polariton branch (UPB, MPB, white solid lines), respectively. The solid red lines show the position of the emitting LPB. The total cavity thickness for the different P-OLEDs from left to right is 238 nm, 248 nm, 258 nm, and 268 nm, respectively.

**Figure S4. Evolution of polariton modes and angle-resolved electroluminescence spectra when increasing cavity thickness in top-emitting design.** Measured angle-resolved electroluminescence spectrum of top-emitting P-OLEDs similar to Figure 4 in the main manuscript, on false-colour scale. The HTL thickness and thus total cavity thickness increases from left to right. The lines indicate the calculated position of the cavity mode (grey dashed lines), excitons (X; grey dash–dotted lines), as well as upper and middle polariton branch (UPB, MPB, white solid lines), respectively. The solid red lines show the position of the emitting LPB. The total cavity thickness for the different P-OLEDs from left to right is 252 nm, 262 nm, 272 nm, and 282 nm, respectively.

**Figure S5.** **Emission spectra of polariton OLEDs at varying operating voltages.** In the measured operating voltage range, we do not observe any polariton-induced spectral shifts or changes in emission linewidth.

**Figure S6.** **Angle-resolved emission intensity of all devices.** All cavity-based OLEDs and POLEDs show slightly sub-Lambertian emission characteristics.

**Figure S7. Simulated Outcoupling Efficiency of POLEDs V1.** The outcoupling efficiency is shown in false colors, while changing the thickness of the ETL and the first HTL (HTL-A, before the SCL, i.e. in the configuration: HTL-A | SCL | HTL-B). While POLED V1a sits close to the predicted outcoupling maximum, the blueshifted POLED V1b shows a slightly reduced outcoupling efficiency.
